# Promoter hypermethylation of *HS3ST2*, *SEPTIN9* and *SLIT2* combined with *FGFR3* mutations as a sensitive/specific urinary assay for diagnosis and surveillance in patients with low or high-risk non-muscle-invasive bladder cancer

**DOI:** 10.1186/s12885-016-2748-5

**Published:** 2016-09-01

**Authors:** Jean-Pierre Roperch, Bernard Grandchamp, François Desgrandchamps, Pierre Mongiat-Artus, Vincent Ravery, Idir Ouzaid, Morgan Roupret, Véronique Phe, Calin Ciofu, Florence Tubach, Olivier Cussenot, Roberto Incitti

**Affiliations:** 1 OncoDiag SAS, Agoranov, Paris, France; 2 Departement of Urology, Saint-Louis Hospital, Paris, France; 3 Departement of Urology, Bichat-Claude Bernard Hospital, Paris, France; 4 GRC-05, University Institute of Oncology, University Paris-6, Paris, France; 5 Department of Urology, Pitié Hospital, Paris, France; 6 Department of Urology, Tenon Hospital, Paris, France; 7 INSERM, ECEVE, UMR 1123, CIC-EC 11425, Paris, France; 8 University Paris Diderot, ECEVE, UMR 1123, Sorbonne Paris Cité, Paris, France; 9 Department of Epidemiology and Clinical Research, Bichat-Claude Bernard Hospital, Paris, France; 10 Computational Bioscience Research Center, KAUST University, Thuwal, Saudi Arabia

**Keywords:** Non-muscle-invasive bladder cancer, Urine-based assay, Genetic and Epigenetic DNA biomarkers, Diagnosis, Surveillance

## Abstract

**Background:**

Non-muscle-invasive bladder cancer (NMIBC) is a high incidence form of bladder cancer (BCa), where genetic and epigenetic alterations occur frequently. We assessed the performance of associating a *FGFR3* mutation assay and a DNA methylation analysis to improve bladder cancer detection and to predict disease recurrence of NMIBC patients.

**Methods:**

We used allele specific PCR to determine the *FGFR3* mutation status for R248C, S249C, G372C, and Y375C. We preselected 18 candidate genes reported in the literature as being hypermethylated in cancer and measured their methylation levels by quantitative multiplex-methylation specific PCR. We selected *HS3ST2*, *SLIT2* and *SEPTIN9* as the most discriminative between control and NMIBC patients and we assayed these markers on urine DNA from a diagnostic study consisting of 167 NMIBC and 105 controls and a follow-up study consisting of 158 NMIBC at diagnosis time’s and 425 at follow-up time. ROC analysis was performed to evaluate the diagnostic accuracy of each assay alone and in combination.

**Results:**

For *Diagnosis*: Using a logistic regression analysis with a model consisting of the 3 markers’ methylation values, *FGFR3* status, age and known smoker status at the diagnosis time we obtained sensitivity/specificity of 97.6 %/84.8 % and an optimism-corrected AUC of 0.96. With an estimated BCa prevalence of 12.1 % in a hematuria cohort, this corresponds to a negative predictive value (NPV) of 99.6 %. For *Follow-up*: Using a logistic regression with *FGFR3* mutation and the CMI at two time points (beginning of the follow-up and current time point), we got sensitivity/specificity/NPV of 90.3 %/65.1 %/97.0 % and a corrected AUC of 0.84. We also tested a thresholding algorithm with *FGFR3* mutation and the two time points as described above, obtaining sensitivity/specificity/NPV values of, respectively, 94.5 %/75.9 %/98.5 % and an AUC of 0.82.

**Conclusions:**

We showed that combined analysis of *FGFR3* mutation and DNA methylation markers on urine can be a useful strategy in diagnosis, surveillance and for risk stratification of patients with NMIBC. These results provide the basis for a highly accurate noninvasive test for population screening and allowing to decrease the frequency of cystoscopy, an important feature for both patient quality of life improvement and care cost reduction.

**Electronic supplementary material:**

The online version of this article (doi:10.1186/s12885-016-2748-5) contains supplementary material, which is available to authorized users.

## Background

Bladder cancer (BCa) ranks among the five most common malignancies worldwide [1]. In a majority of the cases (more than 80 %), Bca is non-muscle-invasive bladder cancer (NMIBC) with low-stage (CIS, pTa, pT1) and low or high-grade [2]. The pTa tumors are associated with a high rate of recurrence (50–75 %) but a low probability (5 %) of progression to lamina propria-invasive (pT1) after resection, whereas carcinoma in-situ (CIS) may be the most common precursor of invasive bladder cancer because it shows a strong tendency to progress (40–50 %) towards muscle-invasive bladder cancer (MIBC) [3]. Cystoscopy and urine cytology are the standard exams for diagnosis and surveillance of NMIBC. The monitoring of NMIBC patients consists of cystoscopic evaluations performed periodically, making it the most expensive of all cancers [4]. The sensitivity of cystoscopy is, however, limited to the tumors that can be identified visually, and the sensitivity of cytology is relatively low in low-stage/low-grade tumors [5]. Therefore, other methods (e.g. NMP22, BTA test, ImmunoCyt and Urovysion) have been developed to reduce the need for cystoscopy with a considerable benefit to both patients and healthcare systems. These noninvasive molecular tests are not recommended for use in diagnosis and monitoring of Bca because of their low diagnostic accuracy [6]. Genetic and epigenetic factors are known to contribute to the occurrence of BCa [7]. *FGFR3* mutations were observed in over 50 % of patients with NMIBC [8] and proposed as a urine prognostic marker for the early diagnosis and detection of recurrences in low-grade tumors [9, 10]. DNA hypermethylation of the CpG islands located in the promoter regions of tumor-suppressor genes has been associated with tumor development in many human cancers [11]. Studies have suggested that measurement of the methylation level in urine sample can aid to early diagnosis of BCa [12–14]. Several recent works showed that the detection of *FGFR3* mutations in combination with methylation analysis could be a promising method for the sensitive detection of primary and recurrent NMIBC [15, 16]. The purpose of this present study is to investigate whether combining methylation measurement of a novel set of DNA methylation markers (*HS3ST2*, *SEPTIN9* and *SLIT2*) to the detection of *FGFR3* mutations can lead to the development a sensitive/specific urine test for the initial diagnosis and the surveillance of low, intermediate, and high-risk NMIBC.

## Methods

### Study design

**Fig. 1:**
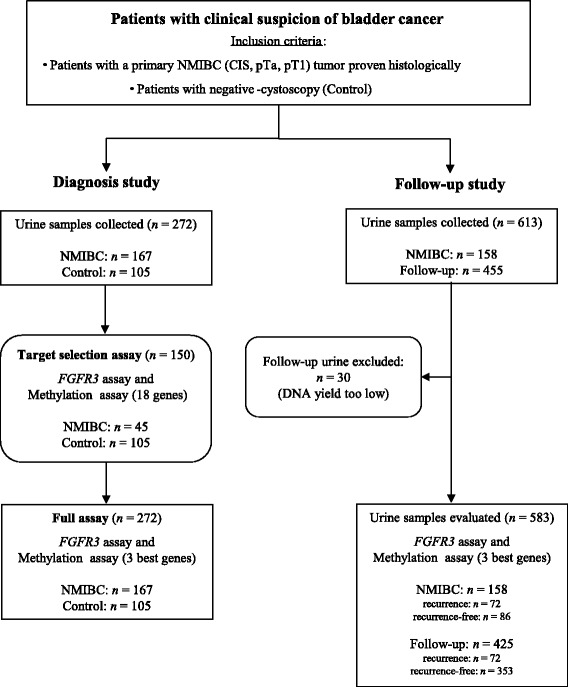
Schematic representation of the study design. For the diagnosis study two series were performed, a Selection set and a Diagnosis set. In total, 272 urine samples were collected. This set consisted of 167 NMIBC patients and 105 controls. For the surveillance study, we included 158 out of the 167 patients who had urine collected in the diagnostic study, and collected a total of 583 urine samples, so having 158 samples at the diagnostic time point and 425 at further time points

As shown in Fig. 1, patients were consecutively and prospectively recruited over a period of time running from 2008 to 2010 on the basis of presenting with a primary NMIBC tumor (pTa, pT1, CIS, low or high grade). As described in [17], NMIBC all patients treated by transurethral resection were eligible, but only those who signed an informed consent were enrolled with a follow-up of 2 years (last follow-up sample collected in 2012). Tumor statuses were histologically confirmed and graded/staged according to the TNM guidelines. A patient was considered as control if the biopsy indicated a benign tissue or, in the absence of a biopsy, if cystoscopy showed no evidence of disease (the controls do not belong to the AUVES project NMIBC cohort). In cases where patients under surveillance were positive for recurrence, they were excluded from the study to be treated. *FGFR3* and methylation assays were carried out independently and as a blind test.

### Urine collection and patients’ information

Urine samples were obtained from a collection code-named AUVES (project reference RECF0998-PHRC 2003) across all 4 participating urology units of public hospitals located at Paris. The samples’ collection and use were reviewed and approved by the Paris Bichat-Claude Bernard hospital ethics committee (approval number: 2004/15). Each urine was collected from the first miction in the morning and before cystoscopy performed on the same day.

One hundred eighty one patients with a primary NMBIC tumor were initially included. We retained 167 of those patients (135 males and 32 females, median age 67 years, range 28–85 years (Fig. 1) as the full set of patients for our study. For the *diagnosis study*, the tumor samples came from all 167 of the full set of patients and the control samples came from 105 other individuals (52 males and 53 females, median age 53 years, range 23–81 years). For the *surveillance study*, we included 158 out of the 167 patients who had urine collected in the diagnostic study (126 males and 32 females, median age 67 years, range 28–84 years), and collected a total of 613 urine samples, so having 158 samples at the diagnostic time and 455 at further time points of the follow-up. The demographic and clinico-pathological patient information is detailed in Tables 1 and 2.

**Table 1:** Clinical characteristics of the 167 patients with initial diagnosis of NMIBC

Characteristic	Case (*n* = 167)	Control (*n* = 105)
Age, yrs.		
Median	67	53
Range	28–85	23–81
Sex, no. (%)		
Male	135 (80.8)	52 (49.5)
Female	32 (19.2)	53 (50.5)
Smoking History, no. (%)		
Current smoker	38 (22.8)	NA
Former smoker	58 (34.7)	NA
Non-smoker	67 (40.1)	NA
Missing	4 (2.4)	
Cytology, no. (%)		
Negative/Positive/Suspicious	123 (73.6)/8 (4.8)/6 (3.6)	105 (100)
Missing	30 (18.0)	
Tumor Stage, no. (%)		
pTa/pT1/CIS	126 (75.4)/25 (15.0)/6 (3.6)	NA
Other	10 (6.0)	NA
Tumor Grade, no. (%)		
Low/High	100 (59.9)/67 (40.1)	NA
Risk Categories, no. (%)		
Low/Intermediate/High	40 (24.0)/56 (33.5)/71 (42.5)	NA

**Table 2:** Clinical characteristics of the 158 NMIBC patients under surveillance

Characteristic	Recurrence-free (*n* = 86)	Recurrence (*n* = 72)
Age, yrs.		
Median	67	66
Range	30–84	28–83
Sex, no. (%)		
Male	74 (86.1)	52 (72.2)
Female	12 (13.9)	20 (27.8)
Smoking History, no. (%)		
Current smoker	25 (29.1)	12 (16.7)
Former smoker	34 (39.5)	22 (30.6)
Non-smoker	26 (30.2)	35 (48.6)
Missing	1 (1.2)	3 (4.1)
Recurrence Rate, no. (%)		72 (45.6)
Time to recurrences, mos.		
Median/Range		10/3–24
Follow-up Urine Analyzed, no.	312	113
Total Urine Analyzed, no.	398	185
Cytology, no. (%)		
Negative/Positive/Suspicious	67 (77.9)/6 (7.0)/2 (2.3)	50 (69.5)/2 (2.8)/3 (4.1)
Missing	11 (12.8)	17 (23.6)
Tumor Stage, no. (%)		
pTa/pT1/CIS	62 (72.1)/14 (16.3)/4 (4.6)	55 (76.4)/11 (15.3)/2 (2.8)
Other	6 (7.0)	4 (5.5)
Tumor Grade, no. (%)		
Low/High	48 (55.8)/38 (44.2)	47 (65.3)/25 (34.7)
Risk Categories, no. (%)		
Low/Intermediate/High	23 (26.7)/25 (29.1)/38 (44.2)	15 (20.8)/28 (38.9)/29 (40.3)

### Processing of urine samples for capture and enrichment of tumor cells

The procedure is detailed in [16]. Briefly, 100 ml of each urine samples were filtered on a nylon membrane of 11 μm of porosity (Millipore) mounted in the corresponding filter holder (Millipore). Each filter was washed with cold 1X phosphate-buffered saline (PBS, pH 7.4) and then removed from the filter holder for DNA isolation. To avoid saturation, urine sample was passed through the filter under gentle aspiration by positive force.

### Urine DNA isolation

DNA was isolated using the QiAmp DNA Mini kit (Qiagen), according to the manufacturer's protocol. The filter is introduced into a sterile tube in presence of AL lysis buffer. The DNA solution was incubated in the presence of proteinase K at 56 °C for at least one 1 h then eluted in 50 μl of elution buffer. DNA concentration was measured using NanoDrop spectrophotometer and stored at −80 °C until uses.

### *FGFR3* mutation analysis

Detection of 4 hotspot mutations of *FGFR3*, namely S249C, Y375C, R248C and G372C, was carried out using allele specific PCR (AS-PCR) in duplex mode (AS-PCR1, AS-PCR2). AS-PCR1 and AS-PCR2 detect simultaneously R248C/G372C and S249C/Y375C respectively, as checked by using the *β-globin* gene included as an internal amplification control. Cycling conditions and concentrations of all primers and probes are as described in [16]. PCR products were separated on capillaries in an automatic sequencer (ABI PRISM 3100 Genetic Analyser, Applied Biosystems). GeneScan Analysis Software (Applied Biosystems) was used for data analysis.

### Identification of best candidate epigenetic markers

We performed literature search to identify hypermethylated genes reported as biomarker candidates to distinguish NMIBC patients from healthy individuals. The search was conducted on the PubMed search engine for the period of time going from 2000 to 2015 using the following search key phrase: (“DNA methylation and/or bladder cancer”) where 499 articles listed. Among these articles, 66 relevant were identified by the following inclusion methylation criteria: 1) They had to be original research studies of the relationship between DNA hypermethylation and bladder cancer; 2) NMIBC cases had to be diagnosed based on histological biopsy; 3) Control subjects had to be free of cancer; 4) Candidate hypermethylated genes had to be determined by microarrays or methylation-specific PCR or quantitative MSP from tissue and/or urine samples. We thus preselected a panel of 18 candidate genes including *COL1A2*, *DDR1*, *DIRAS3*, *DNASE1L*, *EYA4*, *FASTK*, *HS3ST2*, *NPY*, *NTRK3*, *PENK*, *SEMA3B*, *SEPTIN5*, *SEPTIN9*, *SLIT2*, *SYNE1*, *TGFβ1*, *TWIST1*, and *WIF1*.

### Bisulfite DNA modification and methylation analysis

DNA (50 ng) was chemically modified by sodium bisulfite treatment at 50 °C in the dark for 16 h with the EZ DNA Methylation kit (Zymo Research) and eluted in 28 μl of TE buffer (10 mM Tris-HCl (pH 8.0), 1 mM EDTA). To quantify the methylation levels in urine samples, where the DNA amount is often limiting, we used the quantitative multiplex methylation-specific PCR (QM-MSP) using the TaqMan MBG probes technology (Life Technologies), a highly sensitive and specific PCR developed previously by our team [18]. QM-MSP, were carried out in a StepOne Plus Real-Time PCR system (Life Technologies). We used the Universal methylated human DNA standard (Zymo Research) as a calibrator and positive control and urine DNA as sample. QM-MSP reactions were performed in duplicate. In each 20 μL reaction, *HS3ST2*, *SEPTIN9* and *SLIT2* methylated markers and *Albumin* (*ALB*) were amplified with a 1x Kapa Fast Probe (Kapa Biosystems), 400 nM primers and 250 nM TaqMan-MGB probes (Life Technologies). *Albumin* (*ALB*) that not containing CpG sites was used for normalizing the DNA amounts. All primers were designed to have the same annealing temperature (Additional file 1: Table S1). We performed two QM-MSP for co-amplification 6Fam-*SEPTIN9* and Vic-*ALB* (QM-MSP1) and 6Fam-*HS3ST2* and Vic-*SLIT2* (QM-MSP2), respectively. The PCR cycling parameters were initial denaturation at 95 °C for 5 min followed by 95 °C for 15 s, 60 °C for 1 min, repeated 48 times. In supplementary data, we showed that QM-MSP gave similar efficiency (range 99.2–101.4 %) as the quantitative singleplex-MSP (QS-MSP) allowing to reduce of half the number of PCR (Additional file 2: Figure S1). The target’s relative level of methylation (percentage of methylated reference (PMR)) was determined by the “2^-∆∆Ct”^ method were *ΔΔCt = (Ct target - Ct ALB)Control - (Ct target - Ct ALB)Sample*.

### Assays

#### Target selection

We first performed a selection series to select the best methylation targets and to fine-tune our assay, and measured *FGFR3* status and methylation percentage of the 18 genes from the preselected panel on urine DNA from 45 of the 167 NMIBC patients and from the 105 controls. Among those 18 candidate genes, we selected the genes with the following criteria: 1/having at least 1/3 of the control results available and 2/the controls’ mean methylation percent is more than 5 %, and, among those genes, we chose the 3 having the highest specificity for a sensitivity of at least 90 %, so obtaining *HS3ST2*, *SEPTIN9*, and *SLIT2*.

#### Studies

For the diagnosis study, we measured the methylation percentages of *HS3ST2*, *SEPTIN9*, *SLIT2* (Table 3) and the FGFR3 status on the 167 NMIBC patients. For the follow-up study, we assayed the same on the follow-up urine samples. Thirty samples were not assayable because of too low DNA yield. The remaining 583 were from 158 NMIBC patients, with 158 samples at diagnosis’ time and 425 urine samples at various time points of the follow-up. Out of the 425 follow-up urine samples had recurrence and 353 were recurrence-free, defining recurrence as the return of BCa after treatment and after a period of time during which the BCa could not be detected.

**Table 3:** Sensitivity and Specificity of the single or multiple methylation markers in the diagnosis of NMIBC

	% Sensitivity (Se)	% Specificity (Sp)
Diagnosis set (*n* =272)		
*HS3ST2*	82.0 (137/167)	21.20 (22/105)
*SEPTIN9*	90.4 (151/167)	67.6 (71/105)
*SLIT2*	90.4 (151/167)	18.1 (19/105)
*HS3ST2* + *SEPTIN9*	90.4 (151/167)	72.4 (76/105)
*HS3ST2* + *SLIT2*	90.4 (151/167)	34.3 (36/105)
*SEPTIN9* + *SLIT2*	91.0 (152/167)	71.4 (75/105)
*HS3ST2* + *SEPTIN9* + *SLIT2*	90.4 (151/167)	75.2 (79/105)

Note: Performance Se/Sp for either 1/highest Sp with Se > 90 % or 2/highest Sp if no Se > 90 %

### Multivariate analysis and further analyses

#### Diagnosis data

We fit a logistic regression on the data set, using a model with the 3 markers’ methylation values, *FGFR3* status, age and known smoker status at the diagnosis time. *Follow-up data.* We defined: 1/CMI_0 as the CMI at diagnosis’ time (t = 0) and 2/CMI_t as the CMI at monitoring's time (time t > 0). We fit a logistic regression on the whole data set using a model with *FGFR3* status, CMI_0 and CMI_t as defined above. Other models, like including the 3 methylation values separately and/or clinical covariates in the predictors did not show better results for high sensitivity (>90 %). In both computations, we computed ROC curve and AUC, and subsequently optimism-corrected AUC by a bootstrap process with 5000 iterations. We used the ROCR R package [19] in these computations.

We also tested a thresholding algorithm with the *FGFR3* mutation and the sum of the three methylation values at two time points, the diagnosis time and the current time, devised after noting a clustering of recurrence samples in visual inspection in one the corresponding graphs. The details are in Additional file 3.

## Results

### Performance of *FGFR3* assay alone, CMI alone and their combination to detect tumor stage and histological grade

Table 4A represents the association of positive *FGFR3* mutation, together with the methylation status of *HS3ST2*, *SEPTIN9*, *SLIT2*, with low-stage and histological grade for the *initial diagnosis* of NMIBC. The relative figures for *FGFR3* mutation alone in stage/grade tumors were in 46.0 % pTa, 28.0 % pT1, 16.7 % CIS, 30.0 % other tumor stages, 53.0 %/23.9 % low/high-grade. (Other tumors were characterized by pathologists as being NMIBC tumors but not classified as pTa, pT1 and CIS). We observe that S249C is the most relevant mutation in pTa/pT1/low/high-grade, with 69.0 %/42.8 %/62.2 %/66.7 %. The figures for the methylation CMI alone: We identified 90.5 % pTa, 100 % pT1, 100 % CIS, 90.0 % other tumor stages, 89.0 %/100 % low/high-grade. Using the combination of *FGFR3* mutation and methylation, we showed an increase of sensitivity in the low- stage pTa up to 94.4 %, and in the low-grade with 94.0 %. Table 4B shows the same as above for the *surveillance* of NMIBC. The relative figures for *FGFR3* were in 43.6 % pTa, 36.4 % pT1, 50.0 % in other stages, 46.8 %/32.0 % low-/high-grade tumors. As previously observed for the initial diagnosis of NMIBC, S249C is the most relevant mutation detected in recurrence. For concerns the methylation CMI alone: we identified 89.1 % pTa, 90.9 % pT1, 100 % CIS, and 50 % in other stages, 85.1 %/96.0 % low-/high-grade tumors. Using the combination of *FGFR3* mutation and methylation, the detection of recurrence was significantly increased with 96.4 % pTa, 100 % pT1, 100 % CIS, 50.0 % other tumor stages, 93.6 %/96.0 % low-/high-grade tumors. In summary, we showed a strong complementarity between *FGFR3* assay and methylation assay with sensitivity significantly increased for pTa low-grade (Table 4A-B).

**Table 4:** Comparison of sensitivities of *FGFR3* mutations, methylation, and combined markers according to stage and grade

A. Diagnosis study	Primary tumor (*n* = 167)					
Markers	Tumor stage, % (no.)				Tumor grade, % (no.)	
	pTa (*n* = 126)	pT1 (*n* = 25)	CIS (*n* = 6)	Other (*n* = 10)	Low (*n* = 100)	High (*n* = 67)
*FGFR3*						
All mutations	46.0 (58)	28.0 (7)	16.7 (1)	30.0 (3)	53.0 (53)	23.9 (16)
S249C	69.0 (40)	42.8 (3)			62.2 (33)	62.5 (10)
Y375C	13.8 (8)	28.6 (2)		33.3 (1)	17.0 (9)	12.5 (2)
R248C	10.3 (6)	28.6 (2)	100 (1)	33.3 (1)	11.3 (6)	25.0 (4)
G372C	5.2 (3)				5.7 (3)	
S249C/R248C	1.7 (1)				1.9 (1)	
S249C/Y375C				33.3 (1)	1.9 (1)	
Methylation	90.5 (114)	100 (25)	100 (6)	90.0 (9)	89.0 (89)	100 (67)
Combined markers	94.4 (119)	100 (25)	100 (6)	90.0 (9)	94.0 (94)	100 (67)
B. Follow-up study	Recurrence (*n* = 72)					
Markers	pTa (*n* = 55)	pT1 (*n* = 11)	CIS (*n* = 2)	Other (*n* = 4)	Low (*n* = 47)	High (*n* = 25)
*FGFR3*						
All mutations	43.6 (24)	36.4 (4)		50.0 (2)	46.8 (22)	32.0 (8)
S249C	58.3 (14)	100 (4)			54.6 (12)	75.0 (6)
Y375C	20.8 (5)				13.7 (3)	25.0 (2)
R248C	16.7 (4)			50.0 (1)	22.7 (5)	
G372C	4.2 (1)				4.5 (1)	
S249C/R248C				50.0 (1)	4.5 (1)	
Methylation	89.1 (49)	90.9 (10)	100 (2)	50.0 (2)	85.1 (40)	96.0 (24)
Combined markers	96.4 (53)	100 (11)	100 (2)	50.0 (2)	93.6 (44)	96.0 (24)

### Multivariate analysis

#### Diagnosis data

**Fig. 2:**
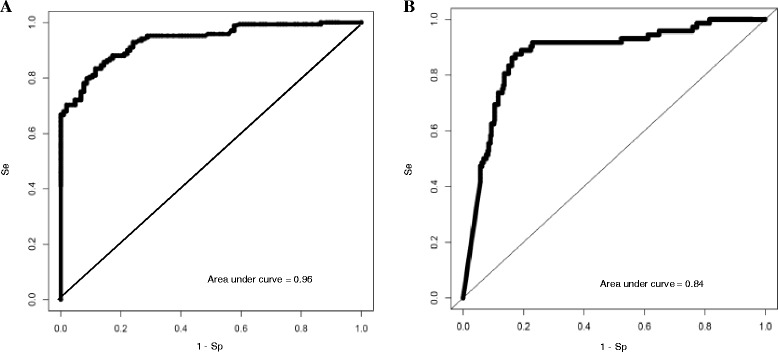
**a** ROC curve of the diagnosis study, using a logistic regression analysis based on presence/absence of *FGFR3* mutation and the CMI of *HS3ST2*, *SEPTIN9*, and *SLIT2*. **b** ROC curve for the recurrence study, using a logistic regression analysis based on *FGFR3* mutation CMI_t and CMI_0

We obtained sensitivity/specificity of 97.6 %/84.8 % and a AUC of 0.97, which resulted in a corrected AUC of 0.96 (Fig. 2a). With an estimated BCa prevalence of 12.1 % in a hematuria cohort [20], this corresponds to a NPV of 99.6 %. *Follow-up data.* We obtained sensitivity/specificity/NPV of 90.3 %/65.1 %/97.0 % and a corrected AUC of 0.84 (Fig. 2b).

### Accuracy of combined test in the surveillance of patients at low-, intermediate- or high-risk NMIBC by using our thresholding algorithm

We obtained sensitivity/specificity/NPV values of, respectively, 94.5 %/75.9 %/98.5 % and an AUC of 0.82 on the whole surveillance set. For concerns risk stratification: As shown in Table 2, 158 patients with first NMIBC were enrolled in the follow-up study and categorized with regard to the risk of recurrence. The patients’ distribution among low/intermediate/high-risk group was 24.0 %/33.6 %/42.4 %. The 72 patients with recurrence were distributed in low/intermediate/high-risk group as 20.8 %/38.9 %/40.3 %. The 68 correctly predicted recurrences were distributed in intermediate/high-risk group as 93.3 %/92.9 %/96.6 %, which again indicates higher propensity of our test to detect high-risk patients.

## Discussion

In this present study, we present a set of markers for a new noninvasive urine testing affording, to our knowledge, the best accuracy for initial diagnosis, surveillance, and risk stratification of NMIBC patients. With a high sensitivity and high NPV, our set of markers is useful for avoiding biopsies and decreasing the frequency of cystoscopic surveillance, thereby allowing for both patient quality of life improvement and cost reduction. Andersson and co-workers showed that the exfoliation of tumor cells into the urine depends on tumor characteristics such as size, stage, grade, and that the filtration of urine samples, as compared to the centrifugation step, increased the diagnostic accuracy of BCa [21]. This observation could be due to the removal of contaminant leucocytes that are smaller size than bladder cells, and this size difference could be exploited to enrich samples for tumor cells. In order to optimize the accuracy of our combined test, we chosen to perform the same procedure of urine samples filtration.

*FGFR3* is a transmembrane tyrosine kinase receptor that binds fibroblast growth factors. Rieger-Christ [22] and Oers [23] showed an overall frequency of *FGFR3* mutations in urine samples for pTa low-grade NMIBC, ranging 43 % to 62 % and confirmed with our two studies (53.0 % in the primary tumor and 46.8 % in detection of early recurrence). As it has been demonstrated by Zuiverloon [24], sensitivity for *FGFR3* assay is correlated with the number of shed low-grade tumor cells. He also showed that low-grade tumors are less likely to shed many cells into the urine as their high-grade counterparts because the high-grade tumors have weaker intercellular attachments with in consequence the difficulty to detect the *FGFR3* mutations. As has been shown by the author, a 24-h collection urines would optimize performance in pTa low-grade tumors and thereby increase the sensitivity of *FGFR3* assay. Consequently, we are likely to have underestimated the *FGFR3* assay sensitivity, performed on a simple urine sample (100 ml). It has been shown that high-risk tumors, generally, have generally more hypermethylated genes than low-risk groups [25]. Consistent with all these observations, we showed the existence of a strong complementarity between detection of *FGFR3* mutations and methylation analysis of *HS3ST2*, *SEPTIN9* and *SLIT2* genes and that their complementarity has afforded the best diagnostic accuracy for low-risk NMIBC. *HS3ST2*, a heparin sulfate sulfotransferase, is expressed predominantly in brain and may play a role in the nervous system [26]. Silencing of the *HS3ST2* gene by promoter hypermethylation has been observed in a variety of cancers [27–29], such as BCa [12]. The *SEPTIN9* gene encodes a member of the conserved septin family of GTP-binding protein that function in key processes including vesicle trafficking, apoptosis, cytoskeletal remodelling and cell division [30]. *SEPTIN9* plays a role in multiple cancers including ovarian, prostate and breast cancer as either an oncogene or a tumor suppressor gene [31]. Hypermethylation of *SEPTIN9* has been observed also in colorectal cancer (CRC) and a commercially available assay for that marker has been developed by the Epigenomics company, affording a sensitivity of 70 % for a specificity of 81 % [32]. As it has been observed for *HS3ST2* methylated gene, we showed its informativeness in NMIBC. The *SLIT* gene family is a recently characterized family of secreted repellents in axon guidance and neuronal migration during the development of the central nervous system [33]. Several studies have showed that *SLIT2*, a tumor suppressor gene, is epigenetically silenced by hypermethylation of the promoter region in many tumors [34–37], and recently in bladder urothelial carcinoma [12]. While the case for informativeness of the methylation and mutation status of well-chosen biomarkers is clearly shown by the literature and our results, we should also mention the considerable potential that the next-generation sequencing (NGS) is showing as compared to previous techniques. This has been highlighted by Ward and colleagues for the detection of low frequency *FGFR3* and TERT mutations in the urine of BCa patients from a few nanograms of DNA [38].

## Conclusions

We showed that our noninvasive urinary test, combining the use of genetic and epigenetic alterations, is at the same time highly sensitive and highly specific in diagnosis, surveillance and can improve risk stratification of NMIBC patients. In the surveillance of NMIBC two directions of use are possible: (1) with low-risk tumors one could reduce the frequency of follow-up cystoscopies, providing a major benefit on the patient’s life quality as well as a positive effect on the medical costs, and (2) with intermediate or high-risk tumors, it could provide earlier detection of tumor recurrence, resulting in improved patient survival.

## Additional files

Additional file 1:
**Table S1.** Oligonucleotides for QM-MSP. (DOC 37 kb) Additional file 2:
**Figure S1.** Efficiency of QM-MSP. (PPT 242 kb) Additional file 3: Prediction algorithm. (DOC 324 kb)

## Abbreviations

ALB: Albumin
AS-PCR: Allele specific-PCR
BCa: Bladder cancer
CIS: Carcinoma in-situ
NMIBC: Non-muscle invasive bladder cancer
NPV: Negative predictive value
MIBC: Muscle invasive bladder cancer
QM-MSP: Quantitative multiplex-methylation specific PCR
