# Supplementary figures and images for: Promoter hypermethylation of HS3ST2, SEPTIN9 and SLIT2 combined with FGFR3 mutations as a sensitive/specific urinary assay for diagnosis and surveillance in patients with low or high-risk non-muscle-invasive bladder cancer
Source: BMC Cancer. 2016 Sep 1;16(1):704. doi: 10.1186/s12885-016-2748-5 (PMC5007990; doi:10.1186/s12885-016-2748-5)

## Slide 1
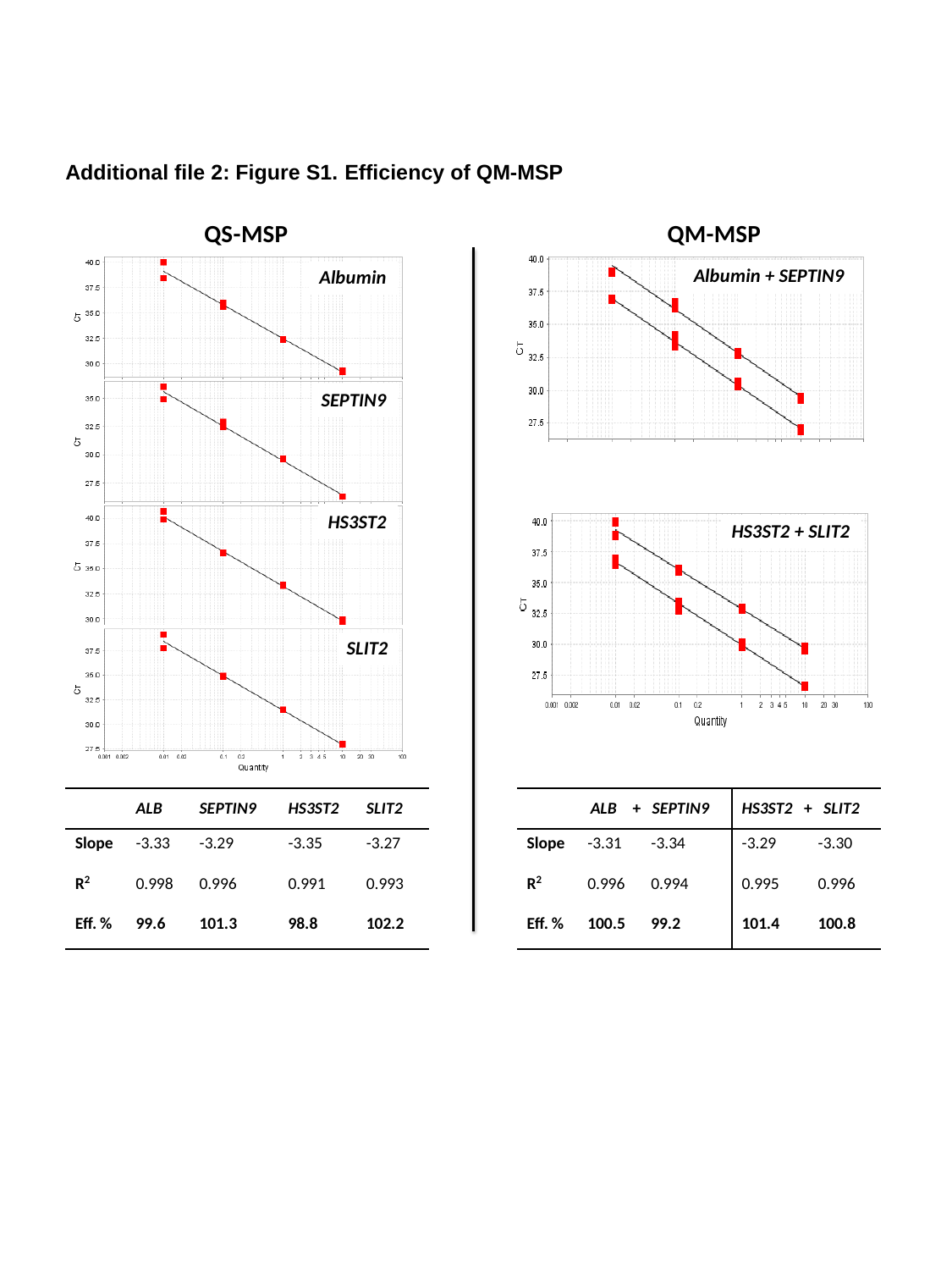

Additional file 2: Figure S1. Efficiency of QM-MSP

Supplement: Additional file 2: — Figure S1. Efficiency of QM-MSP. (PPT 242 kb) [file 12885_2016_2748_MOESM2_ESM.ppt]
