# Supplementary material for: Promoter hypermethylation of HS3ST2, SEPTIN9 and SLIT2 combined with FGFR3 mutations as a sensitive/specific urinary assay for diagnosis and surveillance in patients with low or high-risk non-muscle-invasive bladder cancer
Source: BMC Cancer. 2016 Sep 1;16(1):704. doi: 10.1186/s12885-016-2748-5 (PMC5007990; doi:10.1186/s12885-016-2748-5)
Supplement: Additional file 3: — Prediction algorithm. (DOC 324 kb) [file 12885_2016_2748_MOESM3_ESM.doc]

## **Additional file 3: Prediction algorithm**

THE DIAGNOSIS TEST *Parameters*: we considered 1) Presence/Absence (Positive/Negative) of the *FGFR3* mutation. 2) The sum of the methylation values or CMI (Cumulative Methylation Index) of the three markers at the diagnosis time. *The algorithm*: If *FGFR3* Positive OR (*FGFR3* Negative and CMI > 1.60): Test is Positive. Otherwise: Test is Negative.

THE FOLLOW-UP TEST *Parameters*: Positive/Negative mutation and CMI defined as for the diagnosis study. In addition, we considered 1) The CMI at diagnosis’ time (t=0), which we call CMI_0 and 2) the CMI at monitoring's time (time t>0), which we call CMI_t. 3) Threshold for log(CMI_t)-0.95*log(CMI_0)*(-0.51) to distinguish cancer from normal when the mutation is negative. *Decision criteria*: we note that, in log(CMI_0) *versus* log(CMI_t) plot, recurrence samples appear in two clusters (see Figure below). We fit a linear regression on the upper cluster and used as varying threshold the linear combination so found, namely log(CMI_t)-0.95*log(CMI_0). We chose an optimal value for the threshold (-0.51) and applied the following decision criterium: If *FGFR3* Positive OR (*FGFR3* Negative AND log(CMI_t)-log(0.95*CMI_0)>-0.51 then Test is Positive. Otherwise Test is Negative.
