# Supplementary material for: Promoter hypermethylation of HS3ST2, SEPTIN9 and SLIT2 combined with FGFR3 mutations as a sensitive/specific urinary assay for diagnosis and surveillance in patients with low or high-risk non-muscle-invasive bladder cancer
Source: BMC Cancer. 2016 Sep 1;16(1):704. doi: 10.1186/s12885-016-2748-5 (PMC5007990; doi:10.1186/s12885-016-2748-5)
Supplement: Supplementary file 3 [file 12885_2016_2748_MOESM1_ESM.doc]

Additional file 1: Table S1. Oligonucleotides for QM-MSP

Gene	Oligos	Sequence (5'→ 3')	CpG, no.	Size, bp
Albumin	Forward	GGGATGGAAAGAATTTTATGTT	0	
	Reverse	AAACAAACTAACCCCAAATTCT	0	76
	Probe	Vic-AGGGTTTTTATAATTTA	0	
HS3ST2	Forward	G**CGCG**GGGTTATTTTAGT**CG**	3	
	Reverse	CAAAT**CG**T**CG**CAACAACACAAA	2	110
	Probe	6Fam-**CG**TAAAAA**CG**AAAAACAAC	2	
SEPTIN9	Forward	TTTTTT**CG**T**CG**TTGTTTTT**CGC**	4	
	Reverse	ATC**CG**AATAATCCCATCCAACTAC	1	94
	Probe	6Fam-ATTATGT**CG**GATTT**CGC**	3	
SLIT2	Forward	TAGTTT**CG**T**CG**GGTATTGGGTTT	2	
	Reverse	T**CG**C**CG**ACACTAAAAACTTTCTTTAA	2	80
	Probe	Vic-AGATATTG**CGCG**GTTTT	2	

Note: In underlined bold characters are represented methylated CpG sites
